# Education

**Published:** 1893-10

**Authors:** 


EDUCATION may do much to check evil tendencies, or to develop good ones, but it is a great thing to inherit the right proportion of faculties to start with.—*Items of Interest.*

